# Gastropericardial Fistula After Collis Gastroplasty and Nissen Fundoplication

**DOI:** 10.1016/j.atssr.2023.03.014

**Published:** 2023-04-01

**Authors:** Saranya Prathibha, Ranjan Gupta, Ilitch Diaz-Gutierrez, Amit Bhargava, Rafael Andrade, Madhuri Rao

**Affiliations:** 1 Division of Thoracic Surgery, Department of Surgery, University of Minnesota, Minneapolis, Minnesota

## Abstract

Abnormal fistulization is a rare complication of Nissen fundoplication. A high index of suspicion and timely diagnosis are key factors to guide appropriate management. We describe a 70-year-old woman with a remote history of Collis gastroplasty and Nissen fundoplication presenting with chest pain and a pericardial effusion. She was transferred to our hospital after a pericardiocentesis drained gastric content. She underwent an emergent pericardial window for hemodynamic instability and a left thoracotomy to manage the fistula. We describe the successful management of her gastropericardial fistula and demonstrate that Collis gastroplasty is an additional risk factor for fistulization.

Fistulas are a known complication of Nissen fundoplication and other foregut surgeries. Here, we present a rare case of a delayed gastropericardial fistula for which a Collis gastroplasty performed with a Nissen fundoplication was the likely cause.

A 70-year-old woman with a past medical history of gastroesophageal reflux disease after laparoscopic hiatal hernia repair with Collis gastroplasty and Nissen fundoplication in 2018 presented to an outside hospital with chills, chest pain, and cough in 2021. Workup included computed tomography angiography of the chest, which revealed pericardial gas and effusion in addition to bibasilar pleural effusions. This prompted initial medical treatment of presumed viral pericarditis. Because of lack of improvement and later echocardiography showing concern for tamponade physiology, a pericardial window was performed with temporary drain placement. Scant fluid and loculations were identified. The day after drain removal, she had worsening chest pain, shortness of breath, and worsening pericardial effusion. Esophagography, performed because of her history of antireflux an operation, was negative for a leak. Pericardiocentesis was then performed, which showed brown foul-smelling fluid and radiopaque liquid concerning for gastric contents in the pericardium. In addition, pericardial fluid amylase level was 63 U/L, confirming suspicion of a fistula to the gastrointestinal tract. She was then transferred to our hospital 10 days after her initial admission for further management. She was in atrial fibrillation on arrival and requiring norepinephrine. We took her to the operating room emergently because of the concern for an ongoing leak, sepsis, and cardiac tamponade. The patient was prepared and draped awake before intubation in anticipation of circulatory collapse at induction. During induction, she became hemodynamically unstable, and we immediately performed a subxiphoid pericardial window, releasing gas and fluid. Once she stabilized, we performed endoscopy. We could see bubbling with insufflation through her pericardial window, confirming a connection to the gastrointestinal tract (Figure 1). However, the defect was not visualized, probably because it was within the wrap. We then performed a left thoracotomy to access both the chest and abdomen. Purulent fluid was noted in the pleural space with a rind on the lung as well as a thickened pericardium, suggesting inflammation. After decortication and washout of the left side of the chest, we performed a transdiaphragmatic takedown of her Nissen fundoplication. We opened the diaphragm anteriorly along its attachment to the rib, leaving a cuff for closure. From under the diaphragm, we exposed the fistulous tract just distal to the staple line of the Collis gastroplasty, within the wrap, going into the pericardium (Figure 2). As the defect was small, we performed a gastric wedge resection including the staple line of the gastroplasty and the defect. We then placed a gastrostomy tube for drainage and enteral access. The diaphragm was closed with pledgeted nonabsorbable sutures, and the thoracotomy was closed in the standard fashion.

**Figure 1:**
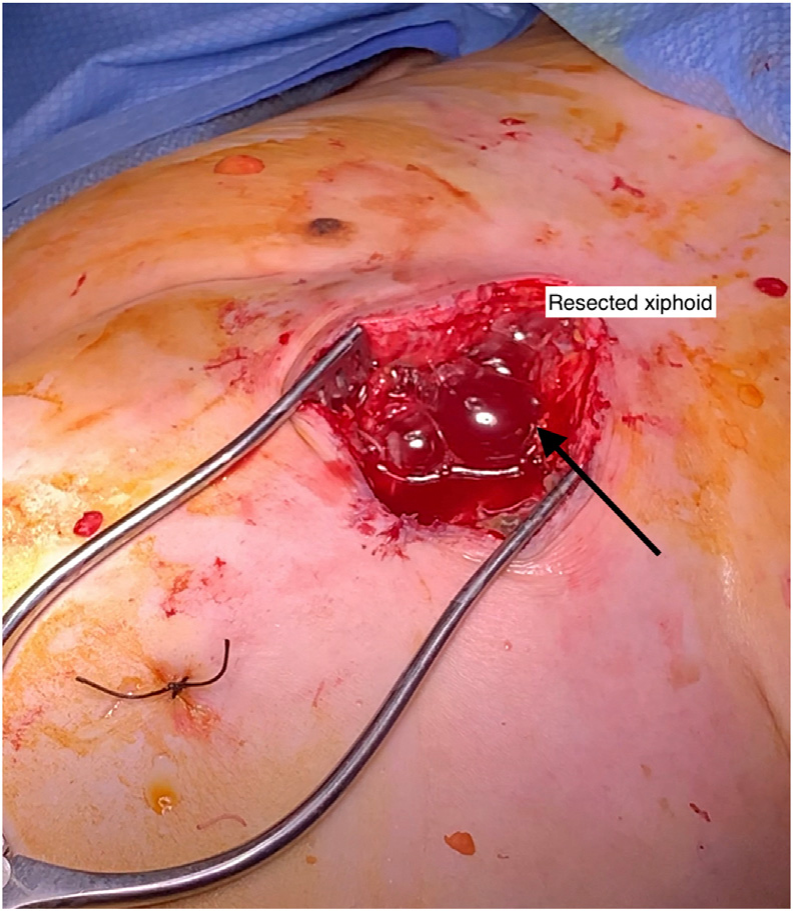
Visualization of bubbling through the pericardial window during endoscopic insufflation signifying a gastropericardial fistulous tract.

**Figure 2:**
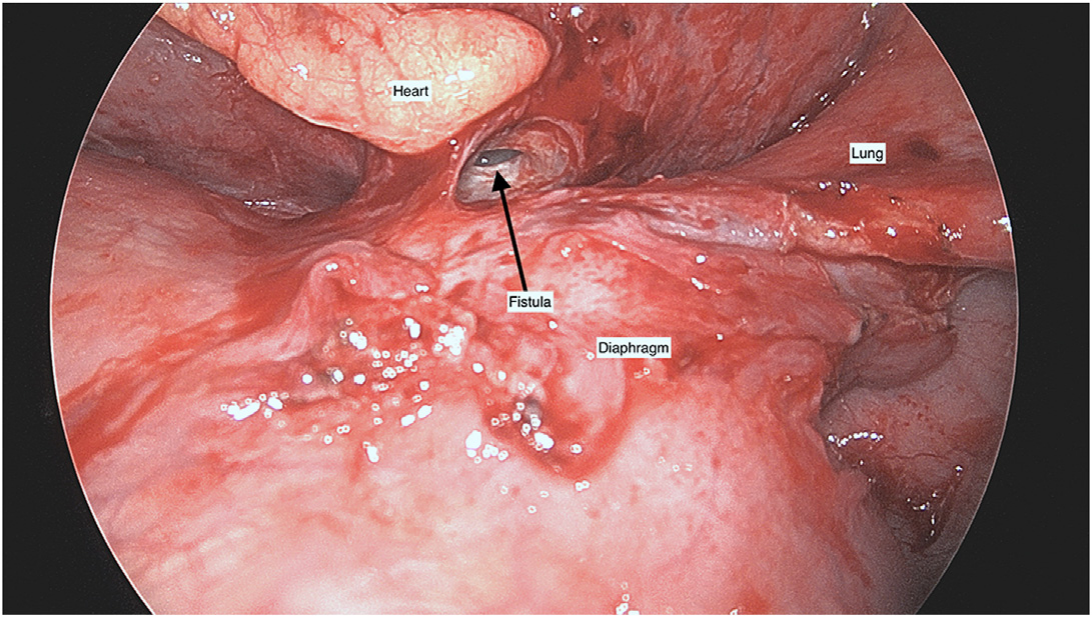
View of the pericardial opening of the gastropericardial fistula with the stomach shown inferiorly.

Postoperatively, the interventional radiologist attempted to transition her gastrostomy tube to a gastrojejunostomy tube for drainage and feeding. However, gastric access was lost, necessitating an emergent return to the operating room for a surgical gastrostomy and jejunostomy tube. She recovered well after this operation. A video swallow and esophagography completed on postoperative day 7 showed no leak. She was started on a clear liquid diet and advanced slowly to a soft diet. Pathologic examination of the gastric wedge showed congestion and focal transmural disruption without dysplasia or malignant transformation at the fistula site. She was subsequently discharged to a long-term rehabilitation facility and eventually to her home. She continues to do well with no reflux issues at this time.

## Comment

Gastropericardial fistulas are a rare complication of Nissen fundoplications. Mortality of gastropericardial fistulas is reported to be >50%.^1^ Davidson and coworkers^2^ performed a literature review and found only 35 cases between 2000 and 2015, most of which were single case reports. Of those, 7 cases were associated with a prior Nissen fundoplication.

The cause behind the formation of these is unclear. However, ulcers, stitch abscesses, and ischemia are included in the differential.^1^^,^^3^ In addition, mechanical factors such as angulation of the lesser curve from the wrap should also be considered.^4^

A common feature specific to these fistulas is their delayed presentation. Kakarala and colleagues^5^ and Sihvo and colleagues^6^ described patients who presented with a gastropericardial fistula 16 years and 7 years after the original Nissen fundoplication, respectively. Diagnosis of these patients remains challenging, resulting in higher chance of death. One reason may be that symptoms are not usually manifested as typical esophageal issues; thus, patients may be seen initially by health care providers unfamiliar with rare complications of prior esophageal operations.^7^

A gastropericardial fistula after a laparoscopic Collis gastroplasty and Nissen fundoplication is exceedingly rare. The fistula was located just distal to the gastroplasty staple line within the wrap, suggesting irritation from the compressed staples. Other risk factors unique to Collis gastroplasty include possible necrosis of the esophageal wall by the heel of the stapler during gastroplasty creation, gastroplasty necrosis, although this is more likely if the left gastric artery is divided for a simultaneous subtotal gastrectomy, and gastroplasty diverticula, which can occur as a late complication. If the wrap or the closure of the hiatus is too tight or if the gastroplasty tube created is too wide, a gastroplasty diverticula can form as a late complication, which theoretically could increase intraluminal pressure and future leak.^8^ No mesh or anchoring sutures to the diaphragm were placed; however, postoperative dysphagia and candidiasis with esophageal erosions, extending her admission, may have been risk factors (Supplemental Figures).

Literature describes treatment of these fistulas with drainage of the pericardium and repair of the stomach with or without omental coverage, similar to our approach.^6^In addition to draining the pericardium, we performed a gastric wedge resection including the gastroplasty staples and fistula. Our management additionally involved a transdiaphragmatic approach to the abdomen. We had good access to both the chest and abdomen without repositioning this critical patient and without needing a traditional thoracoabdominal approach. There was no evidence of a diaphragmatic hernia at 1-year follow-up.

In conclusion, Nissen fundoplication with a Collis gastroplasty can be a risk factor for development of a gastropericardial fistula. A high degree of suspicion and a low threshold for intervention in suspected cases are crucial for a good patient outcome.
