# Supplementary material for: Gastropericardial Fistula After Collis Gastroplasty and Nissen Fundoplication
Source: Ann Thorac Surg Short Rep. 2023 Apr 1;1(3):512–4. doi: 10.1016/j.atssr.2023.03.014 (PMC11708341; doi:10.1016/j.atssr.2023.03.014)
Supplement: Supplemental Figures [file mmc1.docx]

**Supplemental Figures 1 and 2:**

Supplemental Figure 1:

2018 esophagram: Delayed transit and esophageal ulceration

a.


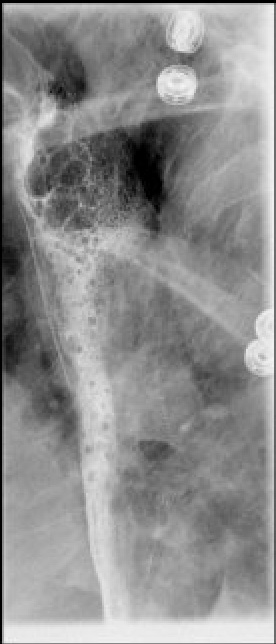


Supplemental Figure 2:

2021 esophagram: esophagram from outside hospital showing no convincing evidence of fistulization


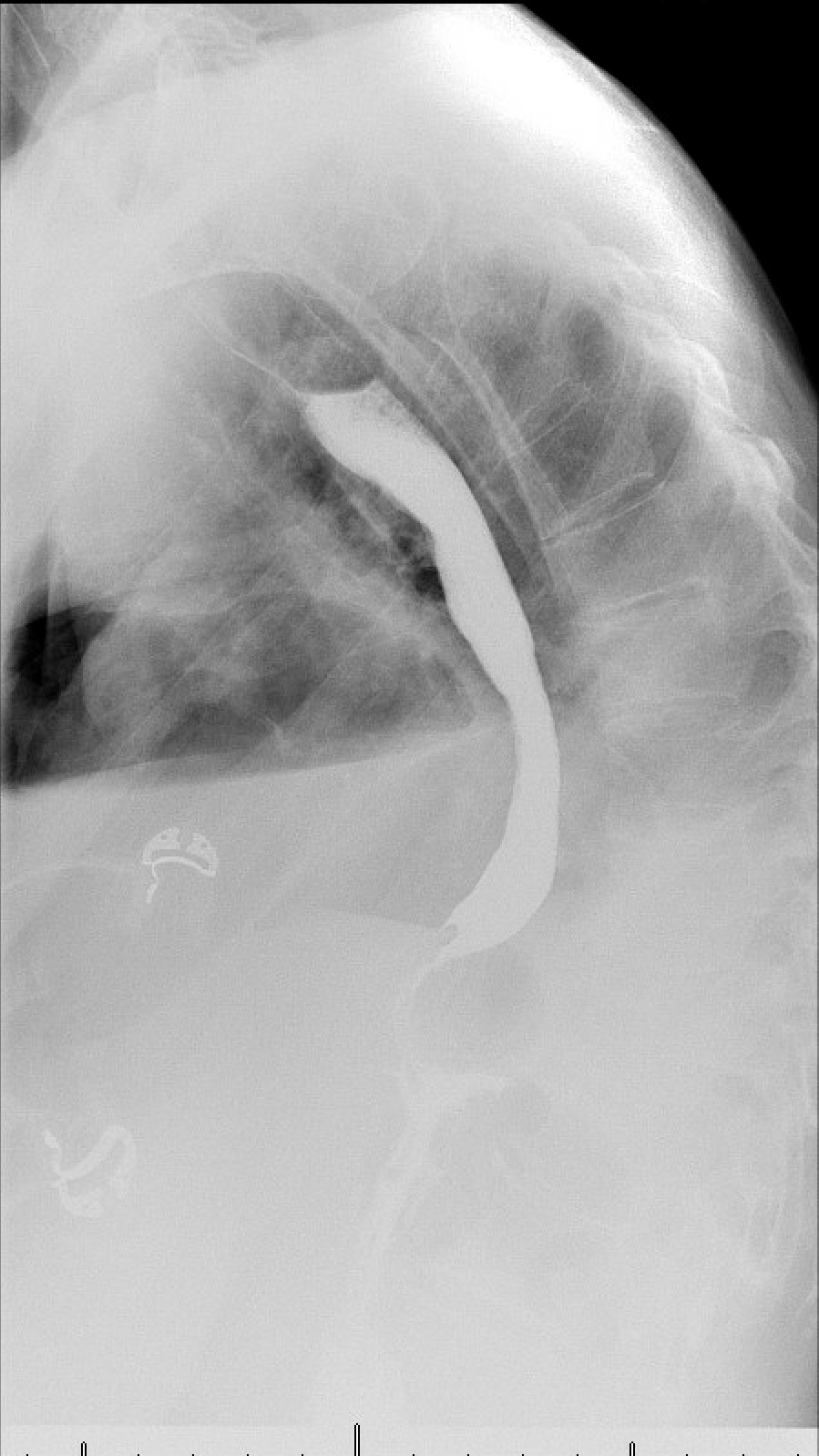

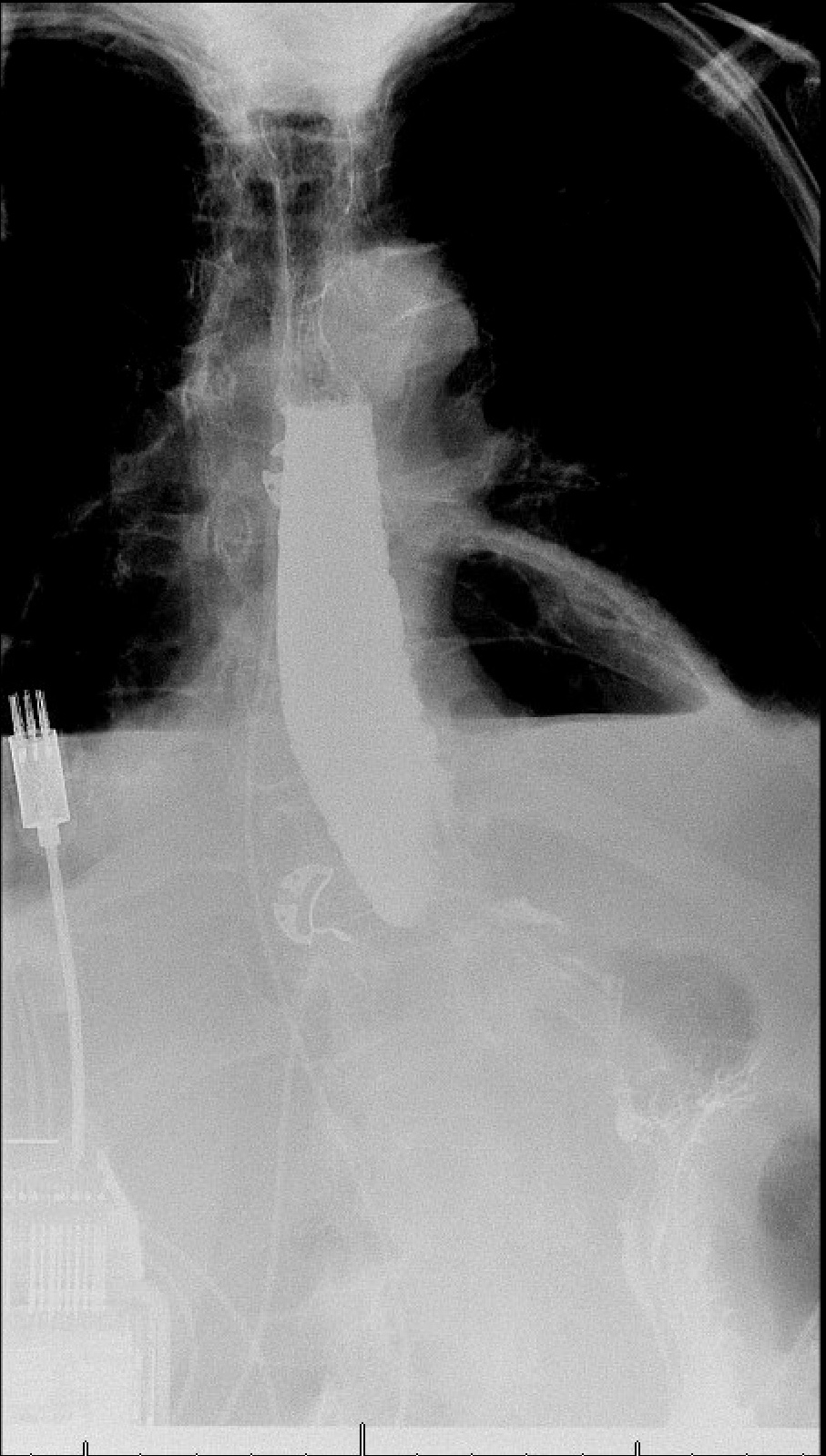
a. b.
